# Lithium as a pharmacological approach to treat behavioral complications in Alzheimer’s disease: a preclinical study

**DOI:** 10.1002/alz.090340

**Published:** 2025-01-03

**Authors:** Monique Patricio Singulani, Rosana Camarini, Leda Leme Talib, Luiz Roberto Britto, Orestes Vicente Forlenza

**Affiliations:** ^1^ Laboratory of Neurosciences (LIM27), Departamento e Instituto de Psiquiatria, Hospital das Clínicas, Faculdade de Medicina da Universidade de São Paulo, São Paulo, São Paulo Brazil; ^2^ Laboratory of Neurochemistry and Behavioral Pharmacology, Departamento de Farmacologia, Instituto de Ciências Biomédicas, Universidade de São Paulo, São Paulo, São Paulo Brazil; ^3^ Laboratory of Neurosciences (LIM27), Departamento e Instituto de Psiquiatria, Hospital das Clínicas, Faculdade de Medicina da Universidade de São Paulo, São Paulo Brazil; ^4^ Laboratory of Cellular Neurobiology, Departamento de Fisiologia e Biofísica, Instituto de Ciências Biomédicas, Universidade de São Paulo, São Paulo, São Paulo Brazil; ^5^ Laboratory of Neuroscience (LIM27), Departamento e Instituto de Psiquiatria, Hospital das Clínicas, Faculdade de Medicina da Universidade de São Paulo, São Paulo, São Paulo Brazil

## Abstract

**Background:**

Nearly all individuals with Alzheimer’s disease (AD) develop neuropsychiatric symptoms (NPS). Lithium is a mood‐stabilizer and is efficient in reducing disruptive behaviors in bipolar‐disorder; this characteristic could be an opportunity to investigate the use of lithium in treating behavioral changes in AD.

**Method:**

We tested lithium carbonate treatment in 3xTg‐AD and age‐matched Wild‐type male mice (CEUA/PROCESS: 1605/2020; 4127240122). The drug was administered ad libitum via lithium‐supplemented chow (1.0g Li_2_CO_3_/kg of chow; 12‐weeks) from 9‐ to 12‐month‐old. Two behavioral test batteries were performed: (1) open‐field test (OFT), novel‐object recognition test (NORT), elevated zero‐maze test (EZMT), and rotarod test; (2) tail‐suspension test (TST), forced‐swim test (FST), and sucrose preference test (SPT). Immunohistochemistry method was performed to analyze amyloid‐β (Aβ) accumulation (6E10) in areas of the brain. Statistical analysis was performed using two‐way ANOVA followed by Bonferroni post‐hoc test and unpaired t‐test (IBM_SPSS Statistics® software 23). We considered statistically significant p<0.05.

**Result:**

Our results demonstrated that lithium had a significant rescue/prevention effect on anxiety‐ and depression‐related behaviors in AD. In both OFT (F_(1, 28)_ = 37.15; p<0.001) and EZMT (F_(1, 28)_ = 19.44; p<0.001), a suppression in anxiety‐related responses was detected in 3xTg‐AD+Lithium mice versus 3xTg‐AD. Similar results were found in TST (F_(1, 28)_ = 11.70; p<0.01) and FST (F_(1, 28)_ = 7.96; p<0.01) with reduction of depressive‐related behavior. In SPT (F_(1, 24)_ = 17.86; p<0.001), 3xTg‐AD+Lithium mice presented an increase in sucrose preference versus 3xTg‐AD, suggesting benefice effects of lithium on anhedonia signs detected in AD. In NORT, 3xTg‐AD+Lithium mice demonstrated greater index in recognition memory parameter versus 3xTg‐AD, suggesting prevention of short‐ (F_(1, 28)_ = 8.37; p<0.01) and long‐term memory (F_(1, 28)_ = 7.99; p<0.01) in AD. We also reported reduction of nearly 50% in Aβ immunoreactivity in areas such as parietal cortex (F_(1, 21)_ = 571.90; p<0.001), CA1 (F_(1, 20)_ = 357.28; p<0.001), dentate gyrus (F_(1, 20)_ = 417.74; p<0.001), subiculum (F_(1, 20)_ = 7073.19; p<0.001), entorhinal cortex (F_(1, 21)_ = 1562.86; p<0.001), and amygdala (F_(1, 22)_ = 419.79; p<0.001), in 3xTg‐AD+Lithium mice versus 3xtg‐AD; suggesting the neuroprotective action of lithium, which partially explains the positive cognitive and non‐cognitive behavioral changes observed with chronic lithium treatment.

**Conclusion:**

The efficacy of a low‐dose lithium treatment in improving cognitive loss and NPS suggest it as therapeutic potential to treat AD.

